# An HIV/AIDS Prophylactic vaccine is possible

**DOI:** 10.1186/1476-8518-5-12

**Published:** 2007-12-19

**Authors:** Qiu Zhong, Ronald B Luftig

**Affiliations:** 1 Department of Microbiology, Immunology and Parasitology, Louisiana State University Health Sciences Center, New Orleans, LA, USA

## Abstract

One needs to think outside of the box, as one of us (Ronald B Luftig) learned from many years as a mathematician, and a biophysicist.

In this short Review, the need to focus on producing high levels of neutralizing antibodies (NAbs) to incoming and conformationally altered virus after it has bound to CD4^+ ^cells is essential.

Increasing the number of gp120 molecules on the surface of L-2 particles, could allow for an enhanced number of NAbs.

The attempt at increasing CD8^+ ^T cell responses in recent vaccine trials has not worked perhaps because it may have allowed HIV to enter into remote sanctuaries. Our approach focuses on increasing NAbs, before high levels of CD8^+ ^T cells are produced.

## Background

It has now become a frequent ritual to read of the newest clinical trial failure and yet the same paradigm goes on [1]. Most recently the promising Phase III trial termed STEP, started in December 2004 was stopped [2]. The strategy was to boost killer T-cells in order to provide a broad-based vaccine and protect high-risk individuals against HIV strains world-wide. An adenovirus vector shuttled 3 HIV genes into the body. Surprisingly, there were more HIV infections in the vaccinees as compared to those in the placebo group [3].

Despite this set-back, trials are starting this Fall using a similar strategy. What is wrong is that vaccinology is not only a science but an art and one needs to take a step back from using the same failed approaches.

**Figure 1:**
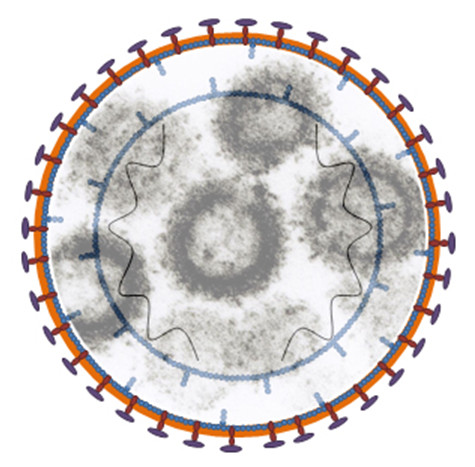
The cartoon of the L-2 particle has 7–10 times more envelope spikes compared to HIV and shows the double membrane of immature gag. Inside the picture is a TEM image of L-2 particles.

**Figure 2:**
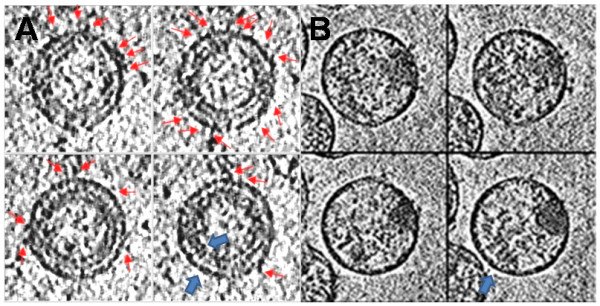
Cryo-EM images: A) The L-2 images show many envelope spikes (red arrows) and a double membrane (blue arrows). B) The control HIV shows few envelope spikes and a single membrane (blue arrow).

We propose that one needs to think differently by presenting defective HIV particles (L-2) which contain 7 to 10-fold more gp120 spikes on their surface in a prime (plasmid pL2)-boost (L-2 particle-Figure 1) strategy. These particles are unique in that they lack several core components of mature virions and present an increased number of gp120 particles (trimeric spikes) as compared to wild type (Table 1). Although mechanistically unclear the increase in surface ENV on these particles is likely due to stabilization of the trimers in the membranes by mutations in the gp41 C-terminus [4] and possibly due to a truncated 56 amino acid NH2 terminal Nef (STOP codon exactly at cleavage site of HIV protease in wild type virus). Preliminary cryo-electron microscopic analysis of L-2 (Figure 2A) relative to HIV (Figure 2B) shows enhanced gp120 spikes (Figure 2A, red arrows) which substantiates the TEM images (Figure 1) and immunoblotting studies that detailed the enrichment of Env on L-2 [5] and pL2 [6]. The double membrane of immature gag in L-2 particles noted by Wright, et al [7] in HIV is also clearly visible (Figure 2, blue arrows). We thank the Lab of Dr. Kenneth Roux for contributing Figure 2, using a cryo-EM method they developed to observe enhanced numbers of spikes on an SIV gp41 mutant [8].

**Table 1:** Compare and contrast L-2 with HIV

**Protein**	**L-2**	**HIV**	**Virion**	**L-2**	**HIV**
Gag	(+)*	(+)**	Genome RNA	(+)	(+)
Pol	(-)	(+)	Infectivity	(-)	(+)
Vif	(-)	(+)	Envelope Spikes	7–10 times	Normal
Vpr	(-)	(+)	Particles	Doughnut like	Normal
Vpu	(+)	(+)	Antigenicity	(+)	(+)
Tat	(+)	(+)	CD4^+ ^Cell Fusion	Yes	Yes
Rev	(+)	(+)			
Env	(+)	(+)			
Nef	(-)	(+)			

* gag uncleaved ** gag cleaved

Therefore we propose that recombinant L-2 particles will be an excellent scaffold for the presentation of native Env trimers. This approach allows for the flexibility of a) building multi-clade Env expressing particles. The new constructs would include Clades C, AE and circulating recombinant forms (CRFs) from China and Africa [9,10] Envs in pL2. Currently, we are testing Clades C and AE to verify that the L-2 backbone for these new Clades still allows for the large number of trimers per particle observed with L-2. If this doesn't work, we will use a different consensus Env expression construct [11] or b) insert structure-based snapshots [12]. It is plausible that these approaches will improve upon current vaccine immunogens by displaying Env trimers in their native context and through stabilized multimerization of Env antigen on L-2 particles.

Simultaneously an intranasal spray is to be administered in order to protect CD4 T-cells in the GALT. The rapid targeting of HIV to the gut mucosa has been well demonstrated in monkeys and recent results show it is only necessary but not sufficient for disease [13].

The properties of L-2 particles are depicted in Table 1 (detailed sequencing manuscript of this data submitted by Zhong, Ikuta and Luftig).

In the model proposed we are thinking about HIV vaccination in a different way. It is important to elicit neutralizing antibodies [14], protect destruction of the GALT and COMBINE this with a potent CTL-based vaccine component. The recent STEP failure further strengthens this triad approach as many T-cell based parameters scored high with the vaccine candidate. We believe this novel approach for elicitation of broadly neutralizing antibodies is the key to its success. However, thus far, no single immunogen has been capable of eliciting a broadly neutralizing response in vivo [11]. Most candidates have relied on mimics of the epitopes recognized by the handful of potent broadly neutralizing antibodies. While reasonable in concept, these approaches have not succeeded.

Several years ago, Drs. Ikuta and Goto discovered a sub-line of HIV-infected cells producing protease deficient defective particles that they called L-2 ([5] and Fig 1). Figure 1 displays a cartoon of L-2 and shows a TEM with several particles containing multiple spikes inside the cartoon. We suggested to Dr. Ikuta that L-2 looked exactly like the doughnut shaped, protease deficient mutants of retroviruses we had studied in the murine system; he considered it was a mutant in RT, since there was no measurable RT activity. Our labs then both sequenced the PR gene nucleic acid from L-2 cells using RT-PCR and found a T nucleotide insertion about one third from the NH2 terminus equivalent of the gene, leading to a STOP codon several nucleotides distant. Thus, there was no PR, RT or INT activity. When the genome of L-2 was sequenced in 1997 it was found that there were 5 mutations [15]. This formed the basis of a patent I received [16]. In 2007 a complete RT-PCR derived DNA sequencing of L-2 particles and its plasmid pL2 was performed. As noted, L-2 particles are unique in that they lack several core components of mature virions. An additional three mutations were found in 2007 (Table 1).

After many years we learned from monkey studies performed by the New England Primate and Harvard groups that mutated SIV will eventually revert back to wild type. However, L-2 particles have 8 mutational differences from HIV (embodied in the 5 differences noted in Table 1). All are related structurally and provide a lock and key effect that might slow down reversions until broad memory B and T-cells responses are generated. Protection of the GALT and provision of a potent CTL-based component would complement these events.

Further, if a high risk individual is infected with a new CRF or Clade other than B, this may be solved by constructing through cassette mutagenesis; multiple new particles based on a backbone of L-2 defective mutant plasmid DNAs mixed together.

AIDS researchers may reasonable argue, that even if multiple broadly neutralizing antibodies did exist, once a high risk patient was infected with HIV, it could avoid neutralization or CTL killing and enter into DNA quiescent T-cells and re-emerge unscathed. However, by an unknown mechanism if L-2 RNA is present within T-cells it serves as a stealth agent and wild type HIV picks up the L-2 envelope with multiple gp120 spikes [17]. This further enhances an opportunity in the body for HIV to be neutralized by pre-existing broadly neutralizing sera or CTL's.

Finally, of great concern is that we don't want to repeat the errors of other excellent AIDS researchers and grow the virus in human T-cell lines, which pick up MHC Class I, II and other components. Thus, L-2 which originated in a human T cell line is being grown in non-human cell lines.

In addition, a recent paper [18] refers to the possibility that if potent T-cell responses are boosted, HIV may move to a sanctuary resulting in persistence of the virus.

The reason for writing this article is not to guarantee an HIV/AIDS vaccine, but to stimulate new thinking for such a vaccine.

## Competing interests

The author(s) declare that they have no competing interests.

## Authors' contributions

RBL drafted MSS. QZ performed experiments related to MSS.
